# Classification of CITES-listed and other neotropical Meliaceae wood images using convolutional neural networks

**DOI:** 10.1186/s13007-018-0292-9

**Published:** 2018-03-23

**Authors:** Prabu Ravindran, Adriana Costa, Richard Soares, Alex C. Wiedenhoeft

**Affiliations:** 1 Department of Botany, University of Wisconsin, Madison, WI 53706 USA; 2 Center for Wood Anatomy Research, USDA Forest Service, Forest Products Laboratory, Madison, WI 53726 USA; 3 Department of Forestry and Natural Resources, Purdue University, West Lafayette, IN 47907 USA; 4 Ciências Biolôgicas (Botânica), Universidade Estadual Paulista – Botucatu, Botucatu, São Paulo Brasil

**Keywords:** Wood identification, Illegal logging, CITES, Forensic wood anatomy, Deep learning, Transfer learning, Convolutional neural networks

## Abstract

**Background:**

The current state-of-the-art for field wood identification to combat illegal logging relies on experienced practitioners using hand lenses, specialized identification keys, atlases of woods, and field manuals. Accumulation of this expertise is time-consuming and access to training is relatively rare compared to the international demand for field wood identification. A reliable, consistent and cost effective field screening method is necessary for effective global scale enforcement of international treaties such as the Convention on the International Trade in Endagered Species (CITES) or national laws (e.g. the US Lacey Act) governing timber trade and imports.

**Results:**

We present highly effective computer vision classification models, based on deep convolutional neural networks, trained via transfer learning, to identify the woods of 10 neotropical species in the family Meliaceae, including CITES-listed *Swietenia macrophylla*, *Swietenia mahagoni*, *Cedrela fissilis*, and *Cedrela odorata*. We build and evaluate models to classify the 10 woods at the species and genus levels, with image-level model accuracy ranging from 87.4 to 97.5%, with the strongest performance by the genus-level model. Misclassified images are attributed to classes consistent with traditional wood anatomical results, and our species-level accuracy greatly exceeds the resolution of traditional wood identification.

**Conclusion:**

The end-to-end trained image classifiers that we present discriminate the woods based on digital images of the transverse surface of solid wood blocks, which are surfaces and images that can be prepared and captured in the field. Hence this work represents a strong proof-of-concept for using computer vision and convolutional neural networks to develop practical models for field screening timber and wood products to combat illegal logging.

## Background

In the last decade, international interest in combating illegal logging has been on the rise (e.g. the US Lacey Act 2008; the Australian Illegal Logging Prohibition Act 2012; the European Union Timber Regulation 2013; Japan’s Act on Promotion of Distribution and Use of Legally Logged Wood Products 2016) as has interest in forensic methods to support them [1–3]. Although emphasis on laboratory-based forensic science is common, especially among laboratory scientists, one of the primary roadblocks to meaningful enforcement of these laws is the availability of efficient field-deployable tools for screening timber outside the laboratory [4]. Conceptually separating laboratory-based forensic analysis of specimens submitted as evidence and field-screening of wood and wood products at ports and border crossings is central to defining the context of the problem to be solved and the degree of specificity necessary to solve it in a way that is meaningful in the real world. Because field law enforcement agents are, in most jurisdictions, required to establish some form of probable cause to detain or seize a shipment of wood, tools intended for field deployment should be designed to meet this need efficiently [4]. The threshold of evidence for probable cause or its international analogs is much lower than forensic-level thresholds, so tools for field screening to establish probable cause can provide results with coarser resolution and lesser certainty than laboratory forensic methods. A typical field screening evaluates the veracity of a claim on a import-export form or shipping manifest. For example, a shipping manifest may claim that the wood is *Khaya* but a field agent determines that the wood is anatomically inconsistent with *Khaya* and in fact is a better match for *Swietenia* and so the shipment could be detained while a specimen is submitted for full laboratory forensic analysis.

This kind of field screening of wood has historically been done, if done at all, by human beings with hand lenses and keys, atlases of woods, or field manuals (e.g.  [5–10] and others). Such keys are based on the fact that wood structure observed macroscopically shows abundant, characteristic variation typically permitting identification at the suprageneric or generic level, with greater specificity possible by highly trained experts or by accessing microscopic characters in the laboratory. Humans with hand lenses are still the state-of-the-art in the field in most countries,^1^ but the time and cost embodied in establishing and maintaining this human-based biological domain knowledge, and the variability of skill and accuracy among those applying such knowledge, means this approach is difficult to scale up to keep pace with increased international interest in and demand for field screening of timber and other wood products.

Computer vision has the potential to provide a practical and cost effective way to replace human-based biological domain knowledge for field screening of wood in trade. One of the primary advantages of this potential is the ability to generate reproducible identifications not dependent on individual human training [11], as long as sufficient images of the woods in question are available for training classifiers and can be captured in the field. In computer vison terms, the problem of image-based wood identification is one of texture-based image classification [12, 13]. Convolutional neural networks have achieved state-of-the-art [14–17] results for image classification in the past few years. While in general convolutional neural networks require large datasets (historically not readily available in the context of wood identification), transfer learning [18] (“Methods” section) provides a pathway to train competitive image classification models using moderate amounts of data by leveraging pre-trained networks, e.g. ones that have been trained on the ImageNet dataset [19]. Convolutional neural networks trained on the ImageNet dataset have been shown to be powerful off-the-shelf feature extractors [20] and transfer learning effectively leverages these general purpose feature extractors, with parameter fine tuning, and permits the use of smaller application-specific datasets for training powerful classifiers. Successfully developing a field-deployable computer vision model for commercial wood species that are threatened or endangered [e.g. species proteted by the Convention on the Trade in Endangered Species (CITES)] is a step toward generating a scalable tool for law enforcement to use to combat global illegal logging.

The botanical issue of species delimitation is not a matter purely of taxonomy when it comes to illegal logging and species conservation through vehicles such as CITES. Any law or treaty that identifies and protects organisms at the species level necessarily depends on the taxonomic circumscription of those species as a foundational predicate for defining the protected organisms themselves. The complex interplay of laws for conservation, taxonomy, species circumscription, and the viability of field-level screening and forensic-level identification of those organisms or their derived products has prompted practical changes to species protection levels in CITES (e.g. the promotion of *Swietenia macrophylla* to be at the same protection level as *Swietenia mahagoni* and *Swietenia humilis* in 2003^2^). Prior to this elevation, unscrupulous traders had the ability to claim a shipment was the less-protected species and forensics could not prove otherwise.

**Fig. 1:**
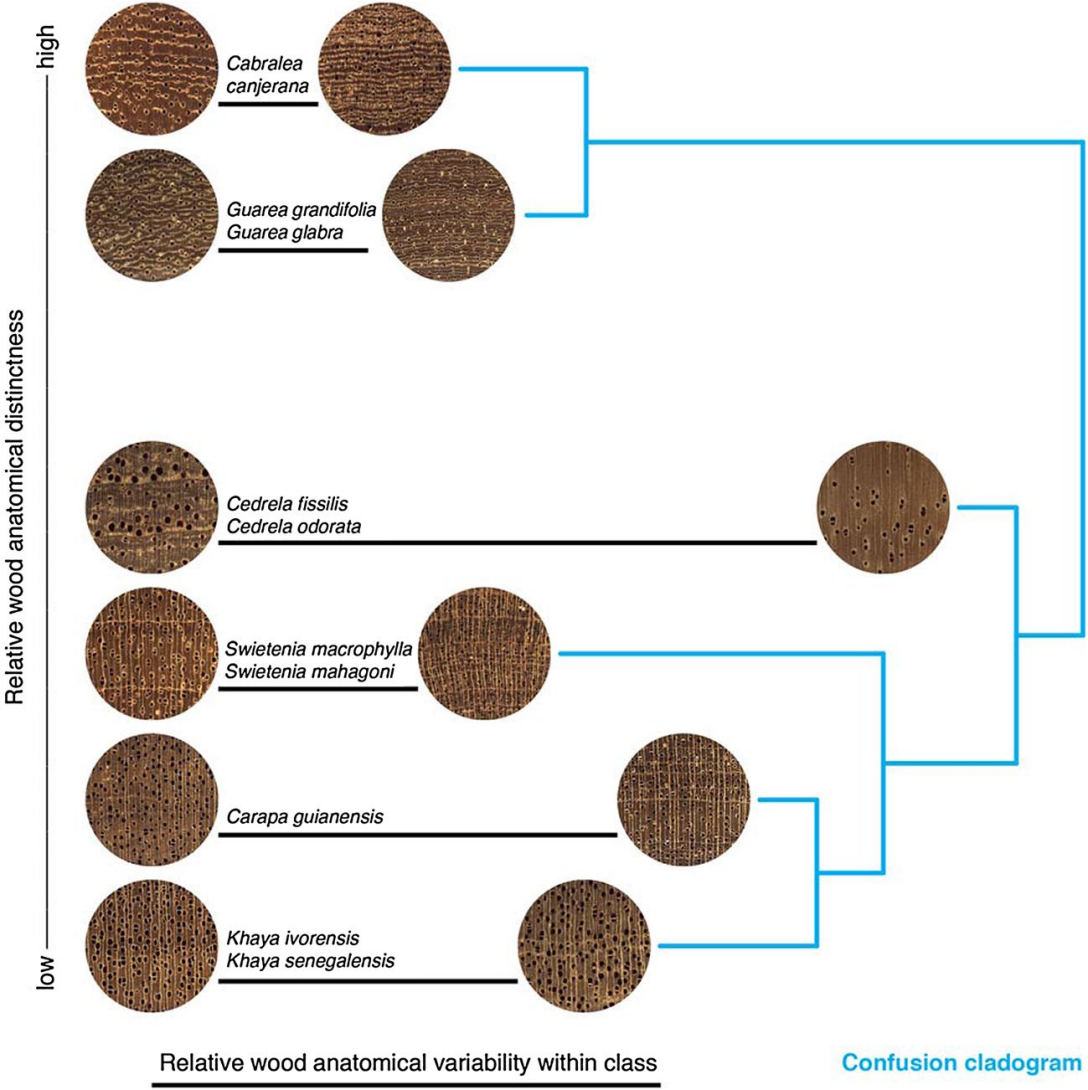
Expected identification relationships based on the generalized wood anatomical distinctness of each group of species (increasing distinctness along the vertical axis) and relative variability within each group of species (variability increasing with increasing bar length along the horizontal axis). The blue tree (confusion cladogram) to the right of the images indicates the expected nested sets of woods likely to be confused with each other based on their anatomical distinctness and variability. Conventional wisdom in wood anatomical identification does not predict species-level resolution

In a real-world practical context, not all woods can or need to be identified to the species level. For example, the trade name African mahogany includes several species of *Khaya* that are frequently sold interchangeably under this trade name and separating them at the species level may not be meaningful in trade—the more important question is likely to be whether they are *Khaya* or the genuine mahogany genus, *Swietenia*. Figure 1 shows a “confusion cladogram”, a depiction of the expected nested likelihoods of woods (at the genus level) that could be confused with each other based on traditional hand lens wood identication. The relative anatomical distinctness of each genus (vertical axis) and the relative variability within the genus (extent of the black bars along the horizontal axis) are provided as representations of traditional wood identification domain knowledge. Based on the relationships in Fig. 1, *Khaya* and *Swietenia* would be expected to be somewhat confusable, despite being fundamentally different woods with different commercial values, different wood technological properties, and different level of protection under CITES. A field-screening technology that could determine the genus of a wood in trade would be of great practical value, with one that could provide a reliable species-level discrimination being the idealized goal.

In this study we report on highly effective computer-vision classification models, based on deep convolutional neural networks trained via transfer learning, to identify 10 neotropical species in the family Meliaceae, including CITES-listed species *Swietenia macrophylla, Swietenia mahagoni*, *Cedrela fissilis*, and *Cedrela odorata* [7]. We selected taxa that have real-world relevance in international timber trade and/or represent an interesting range of overlapping (inter- and intra-class variability) wood anatomical patterns, structural variability, and distinctness of anatomical pattern at multiple scales (Fig. 1). These models discriminate the various woods based on digital images of the transverse surface of solid wood blocks, using images roughly at a hand lens magnification, thus also suitable for human-mediated provisional identification. The transverse surface of wood specimens at a port, border crossing, or other point of control can be prepared for imaging with a modicum of training and a sharp utility knife. We demonstrate proof-of-concept for image-based wood identification using convolutional neural networks and suggest avenues of future inquiry, to develop and eventually deploy computer vision in the field.

## Methods

### Convolutional neural networks

Convolutional neural networks (CNNs) [23] are state-of-the-art classifiers [14–17] that have powered many recent advances in image classification. CNNs have a multilayer architecture of convolutional operations interspersed with non-linear activation functions and pooling operations which enable them to learn rich non-linear representations for image classification. The parameters of CNNs can be learnt automatically in an end-to-end fashion given sufficient data. While automated representation learning from data is an attractive feature, training CNNs from scratch typically requires large datasets which may not be available. A practical way to build CNN based image classifiers using moderately sized datasets is through transfer learning where features learnt using large datasets in a related domain are leveraged for the task at hand.

### Transfer learning

Transfer learning [18] is a machine learning technique for building powerful classifiers when large datasets are unavailable. In transfer learning, knowledge gained by training accurate classifiers (pre-trained models) using large datasets in one domain is reused/leveraged to build powerful classifiers in a related domain where access to large datasets is unavailable. In the context of image classification using CNNs, the layers closer to the input layer learn generic features such as edges and blobs. Transfer learning effectively exploits this observation and enables building powerful CNN based image classifiers using moderately sized datasets. Specifically, the lower layers (close to the input) are retained along with their learned parameters; whilst the top layers are removed/customized for the problem at hand and initialized with random parameters. *All* the parameters of this customized network are learnt using the available dataset and this process is called *finetuning*. The VGG16 [15] model pre-trained on the ImageNet dataset [19] is well studied for image classification via transfer learning and we employ it to build classifiers for wood identification.

### CNN architecture for wood identification

**Fig. 2:**
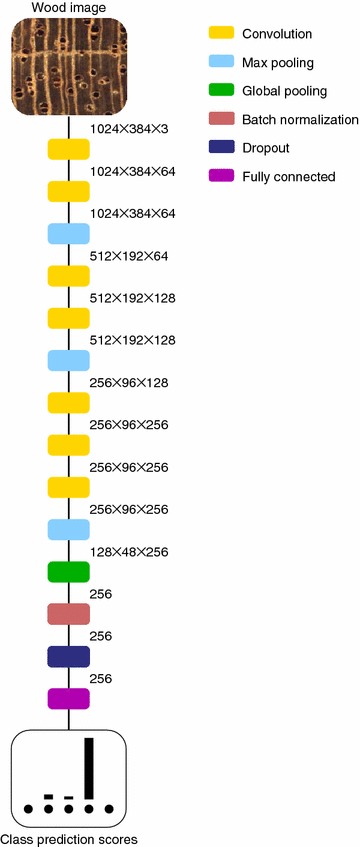
A schematic of the CNN architecture employed for wood identification. We trained models with both global average pooling and global max pooling layers (with the performance being comparable). The dimensions of the feature maps are in pixels of the form: (height, width, depth). The final classification layers has 10 and 6 outputs for the species and genus level models respectively

The architecture for the CNN image classifier that we trained for wood identification is shown in Fig. 2. We used the first 10 layers (7 convolutional and 3 max pooling layers) from the pre-trained VGG16 network. All the convolution layers have $3\,{\text{ pixel }} \times 3\,{\text{ pixel }}$ kernels and ReLU activations [24], with a one pixel wide zero padding such that the output feature maps of each convolution layer has the same dimensions as its input. The max pooling layers in the VGG16 architecture pool data over a $2\,{\text{ pixel }} \times 2\,{\text{ pixel }}$ image window and have stride 2 pixels, which results in halving the dimensions of the input feature map to the layer. We add global pooling (two variants), batch normalization [25], dropout [26] and fully connected layers on top of the 10-layers of the VGG16 base. The global pooling layer provides a measure of the “energy” in each of the texture channels that are extracted by the fine tuned VGG16 convolution layers. We trained models with global average pooling and global max pooling layers. We used a dropout value of 0.5. The fully connected layer produced class prediction scores for 10 and 6 classes for the species and genus level classification models respectively. Softmax activation was used to output class prediction scores in the fully connected layer.

### Specimen preparation and imaging

Taxa selected for the study (Table 1) represent the more common commercial and confusable neotropical Meliaceae woods, as well as representative species of *Khaya*, as this genus is grown in plantation in some neotropical areas. Complete transverse surfaces of scientific wood specimens from the xylaria^3^ at the US Forest Products Laboratory in Madison, WI were sanded for macroscopic imaging. $2048\,{\text{ pixel }}\times 2048\,{\text{ pixel }}$, 8-bit RGB images of the transverse surfaces (representing $\sim 6.35{\text{ mm }} \times 6.35{\text{ mm }}$ of tissue) were captured using a Point Grey Flea 3 digital camera (FL3-U3-88S2C-C) without image sharpening, and optimizing the camera shutter times to center the image histogram around 128 whilst minimizing the number of overexposed and underexposed pixels. When possible, more than one unique image was collected from each xylarium specimen. After image capture, we annotated the images to indicate the presence of surface preparation artifacts, atypical wood anatomy, misidentified wood specimens, and to designate archetypal specimens. This resulted in a total of 2303 images.

**Table 1:** Training and testing splits of the image dataset by class at the species level

Species	Training split	Testing split
*Cabralea canjerana*	41	18
*Carapa guianensis*	305	134
*Cedrela fissilis*	133	59
*Cedrela odorata*	354	160
*Guarea glabra*	45	20
*Guarea grandifolia*	33	14
*Khaya ivorensis*	240	105
*Khaya senegalensis*	36	16
*Swietenia macrophylla*	372	165
*Swietenia mahagoni*	37	16
Total	1596	707

Splits for the genus level model are the sums of the individual species in each genus. The total number of images is 2303

### Patch dataset creation

We divided the dataset of 2303 images into a (approximate) $60\%/40\%$ train/test split. The summary of the training and testing split image counts are provided in Table 1. Next, patches of size $2048\,{\text{ pixel }} \times 768\,{\text{ pixel }}$ were extracted from the dataset images and resized to $1024\,{\text{ pixel }} \times 384\,{\text{ pixel }}$. For each class (species), we extracted 500 and 200 patches from the training and testing splits respectively. Due to the classes not being balanced in our dataset, we allowed considerable overlap between patches for classes with fewer images. Such minority class oversampling has been shown to be effective for training CNNs in the presence of class imbalance [27]. We also created a dataset to train/evaluate the genus-level classifier by taking a subset of 500 training patches and 200 testing patches from the above patch dataset in such a way that the species image proportions within a genus was respected. The summary of the number of patches used for training and evaluating the species and genus level models are in Table 2.

**Table 2:** Summary of patch datasets for species/genus level models

Model	#Classes	#Training patches	#Testing patches
Species level	10	5000	2000
Genus level	6	3000	1200

### Training

**Fig. 3:**
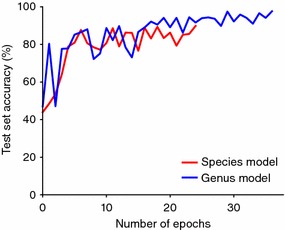
Plot of patch-level prediction accuracies for the species and genus models during training. Accuracies are shown up to the epoch at which early stopping was done (epoch 25 for the species model and epoch 37 for the genus model)

Model training was carried out in two phases. In the first phase, we used the convolutional layers of the VGG16 network as feature extractors (i.e. layer weights frozen) and the custom top level layers were trained for 30 epochs using stochastic gradient descent with a learning rate of $10^{-4}$ and a momentum of 0.9. In the second stage we finetuned the parameters of the entire network, including the convolutional layers, for 100 epochs with early stopping if the test split accuracy did not improve for 10 epochs. The Adam optimizer [28] was used for the second stage with a learning rate of $10^{-3}$ and a decay of $5\times 10^{-4}$. For both stages we minimized the categorical cross entropy loss using a batch size of 8. The architecture definition and training was implemented using Keras [29] with the TensorFlow [30] backend on a NVIDIA Titan X GPU. Accuracy curves, for the second stage of training, are presented in Fig. 3.

### Evaluation

**Fig. 4:**
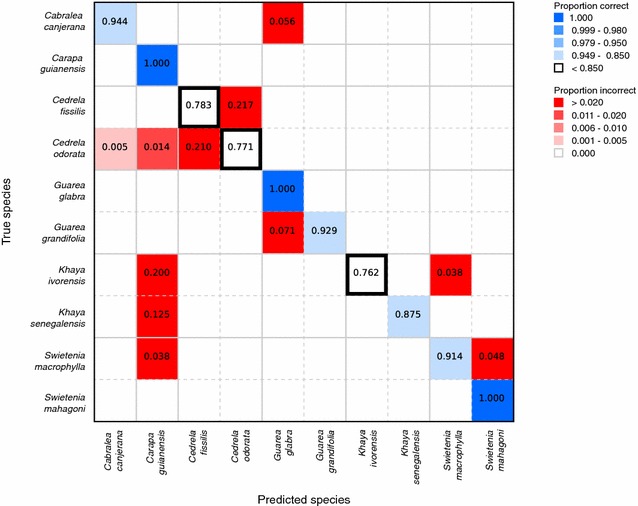
Image-level confusion matrix for the 10-class species-level model. On-diagonal results (correct predictions) coded in tones of blue, with proportions in bold. Off-diagonal results (incorrect predictions) coded in tones of red, with values of zero not presented or colored

**Fig. 5:**
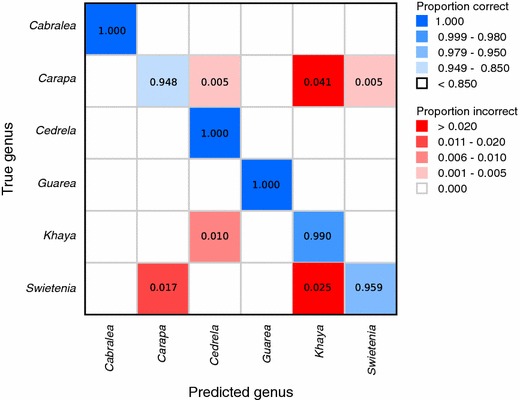
Image-level confusion matrix for the 6-class genus-level model. On-diagonal results (correct predictions) coded in tones of blue, with proportions in bold. Off-diagonal results (incorrect predictions) coded in tones of red, with values of zeros not presented or colored

Accuracies of class predictions on the patches in the test split are reported in Table 3. In addition, for the images in the test split, we extracted 5 equally spaced patches from each image, summed the prediction scores for these 5 patches and chose the class with the maximum summed score as the prediction for the image. The image level accuracies are also presented in Table 3. To understand the errors made by the models we provide confusion matrices for the species and genus models at the image level (Figs. 4, 5). We present the confusion matrices and training curves for the models with the global average pooling layer (the corresponding entities for the model with the global max pooling layer were comparable and are not presented).

**Table 3:** Model prediction accuracies

Model	Patch level (%)	Image level (%)
Global average pooling		
Species level (10 class)	89.8	87.4
Genus level (from 10-class species level)	95.4	95.4
Genus level (6 class)	97.6	97.5
Global max pooling		
Species level (10 class)	89.2	88.7
Genus level (from 10-class species level)	96.9	97.0
Genus level (6 class)	97.2	97.3

## Results and discussion

Wood anatomy typically varies characteristically at the generic rather than the specific level even when analyzed with light microscopy [31]—species-level distinctions are typically based on external morphological, reproductive and vegetative characteristics that are not reflected in the wood anatomy, at least as analyzed by human experts. Given this traditional limitation of wood identification, it is necessary to distinguish between species-level and genus-level accuracy and hence we trained and evaluated 10-class species-level and 6-class genus-level models.

The overall accuracy of the predictions of our models is shown in Table 3. In order to calculate the genus-level accuracy from the 10-class species-level model (shown on the second row of Table 3 (“Genus level (from 10-class species level)”), we consider predictions of the wrong species but the correct genus as correct predictions and report those metrics. The image-level confusion matrices for the species-level and genus-level models are shown in Figs. 4 and 5 respectively.

### 10-Class species-level model

Slightly less than $6\%$ of the images of *Cabralea* were misclassified as *Guarea*, and within *Guarea*, approximately $7\%$ of the images of *Guarea grandifolia* were misclassified as *Guarea glabra*, but no images of either genus were classified as any genus outside these two. As shown in the confusion cladogram of Fig. 1, these results are in keeping with expectations based on traditional wood identification, and represent sensible errors.

The predictions made by the model for *Carapa* images are perfect, but the class also draws misclassified images from four species of three genera, which is again consistent with the known high variability of *Carapa*, as a taxon, as shown in Fig. 1, where the horizontal bar indicating variability is second only to that for *Cedrela*.

Within *Cedrela*, the genus identified as the most variable in Fig. 1, all the misclassified images (more than $20\%$) of *Cedrela fissilis* are predicted as *Cedrela odorata* and all the misclassified images (also more than $20\%$) of *Cedrela odorata* images are predicted as *Cedrela fissilis*. For *Cedrela* the model correctly determines the genus, but these CITES-listed species cannot be as reliably separated from each other as other species in our dataset. The absence of non-CITES-listed *Cedrela* in our dataset precludes the possibility of testing the ability of our model to discriminate between CITES-listed and non-CITES-listed species in this genus.

The model showed comparatively poor performance in classifying images of both species of *Khaya*, both in terms of the relatively low proportion of images correctly classified, and in that all misclassified images were assigned to species in other genera. Nearly all those images were attributed to *Carapa guianensis*, which is the closest nested relationship shown in the confusion cladogram (in Fig. 1), the remaining were classified as *Swietenia*, the next most closely related group in the cladogram.

Within *Swietenia*, the model’s classification of *S. mahagoni* images was perfect, but slightly less than $4\%$ of *S. macrophylla* images were classified as *Carapa guianensis* and nearly $5\%$ were incorrectly classified as *S. mahagoni*. Interestingly, no images of *Swietenia* were classified as *Khaya* or *Cedrela*.

When these species-level model results are reconsidered at the genus level, all the predictive errors within *Cedrela* and *Guarea* disappear, and less than $2\%$ of *Swietenia* and less than $1\%$ of *Cedrela* images are misclassified outside their genera. Because all the misclassified images of *Khaya* were attributed to species in different genera, consolidating the species-level results at the genus level does not alter the model’s relative performance in this genus.

### 6-Class genus-level model

Field screening of wood for most law enforcement purposes need not be accurate at the species level. Hence we also created an explicit genus level model in order to determine if clubbing species of the same genus into a single generic class would increase genus-level performance.

Table 3 presents summary data showing the improved performance of the explicit 6-class genus-level model compared to the genus-level results from the 10-class species-level model. The 6-class genus-level model (Fig. 5) shows major improvement for *Cabralea*, *Cedrela*, and *Guarea*, all of which are classified perfectly, and for *Khaya* which has only $1\%$ of its images misclassified (as *Cedrela*). Interestingly, *Carapa*, despite being monotypic in the 10-class species-level model (and thus functionally a genus-level class in that model), loses specificity in the 6-class genus-level model, with approximately $4\%$ of its images classified as *Khaya*, and another half-percent each as *Cedrela* and *Swietenia*. Roughly $2\%$ of the *Swietenia* images are classified as *Carapa*, and roughly the same amount are classified as *Khaya*. This is interesting because in the 10-class species-level model, the only misclassification of a *Swietenia* image outside the genus was as *Carapa*. These results suggest that future work may benefit from targeted clubbing of some classes, especially if the real-world utility of species-level identification during field screening is minimal or non-existent.

In addition to achieving a useful level of resolution for field identification of wood specimens in trade, clubbing the individual species within each genus into one class has several potentially favorable side-effects. If one has access to expert-level biological domain knowledge about class variability in the dataset, targeted decisions on label space granularities can result in classes that are more favorable for training supervised machine learning algorithms [32]. Lack of access to sufficient reference images at the species level is likely to be endemic and a limiting factor for image-based wood identification, but classes clubbed to the genus level are more likely to contain sufficient images. In addition to the biological and machine learning considerations and constraints, access to law enforcement expertise could further inform class definition taxonomies to ensure that the ultimate field-level tool is most relevant in the locales it is deployed.

## Summary

The global context of trade in illegally logged wood necessarily invokes the need for large-scale or scalable solutions. Enforcement of existing law and support for additional protection requires a scientific and forensic basis for evaluating claims about wood and wood products, whether that claim is a species, a genus, a region of origin, or age. One part of a global solution is laboratory-based forensic methods that support successful prosecutions, but it is first necessary for law enforcement to identify, detain, and sample problematic shipments at points of control using effective field screening tools.

We presented a deep convolution neural network, trained using transfer learning, capable of separating anatomically similar commercial and endangered woods of the Meliaceae family at both the genus and species level, with image-level accuracy greater than 90%. This accuracy is far in excess of the minimum necessary to establish probable cause or other appropriate legal predicate for seizing or halting the transport of a shipment of wood. Our models operate on macroscopic images of the transverse surface of wood blocks—such a surface can be prepared and an image taken *in situ* by trained field agents. Convolutional neural networks trained end-to-end, either using transfer learning or trained from scratch (given sufficient datasets), clearly have the potential to provide a scalable way to accommodate model building in the various controlled contexts. Although we used the well-studied VGG16 pre-trained network to build our models, we are currently exploring other model architectures (e.g. [16, 17]). These alternate architectures, and their variants, have fewer parameters than the VGG networks and maybe well-suited for a system that can be deployed using mobile phones [33]. We are also exploring scaling the models to hundreds of woods with human expert-informed label space taxonomies, and are studying methods to visualize [34, 35] and interpret the representation learned by the deep neural networks and compare it against traditional human-designed identification keys.

We believe that deep convolutional neural networks along with expert-informed label space taxonomies for controlling context show promise in developing an effective field screening tool for wood identification. For computer vision solutions to contribute most robustly in this area, either the context must be tightly controlled so that the number of classes remains low (e.g. a regional port with a limited number of local taxa) or the models must scale-up beyond the proof-of-concept we present here, by discriminating $10^2$–$10^3$ classes of wood successfully, and such models must be tested and vetted in field application. The cooperation of machine learning experts, law enforcement officers, and forensic wood anatomists shows great potential to develop informed label space granularities that ensure the most relevant field-deployable models for field screening wood identification. Models developed, tested, and vetted cooperatively in this way can provide reliable, scalable field-screening of wood in trade to protect threatened and endangered species (e.g. CITES-listed species) and combat illegal logging.

## Footnotes

1 Note that in Brazil, the state of São Paulo’s Instituto Florestal developed an “online” identification system where field agents transmitted macroscopic photos to experts in the laboratory who provided near real-time identifications to inform detention decisions.

2 The polymorphic nature of *Swietenia* and its generic circumscription are considered in detail in [21, 22].

3 The Madison (MADw) and Samuel J. Record (SJRw) collections were used.

## References

[1] Wiedenhoeft A, Baas P, editors. Wood science for promoting legal timber harvest. IAWA J. 2011;32(2):121–296.

[2] Dormontt EE, Boner M, Braun B, Breulmann G, Degen B, Espinoza E, Gardner S, Guillery P, Hermanson JC, Koch G, Lee SL, Kanashiro M, Rimbawanto A, Thomas D, Wiedenhoeft AC, Yin Y, Zahnen J, Lowe AJ (2015). Forensic timber identification: it’s time to integrate disciplines to combat illegal logging. Biol Conserv.

[3] Lowe AJ, Dormontt EE, Bowie MJ, Degen B, Gardner S, Thomas D, Clarke C, Rimbawanto A, Wiedenhoeft A, Yin Y, Sasaki N (2016). Opportunities for improved transparency in the timber trade through scientific verification. BioSci.

[4] United Nations Office on Drugs and Crime: Best practice guide for forensic timber identification. 2016.

[5] Chalk I. Identification of hardwoods: a lens key. Forest Products Research Bulletin No. 25, USA. 1952.

[6] Ilic J. The CSIRO macro key for hardwood identification. Highett, Victoria, Australia: CSIRO. 1990.

[7] Miller R, Wiedenhoeft A. CITES identification guide—tropical woods: guide to the identification of tropical woods controlled under the convention on international trade in endangered species of wild fauna and flora. An Initiative of Environment Canada. 2002.

[8] Coradin VTR, Camargos JAA, Marques LF, Silva-Junior ER (2009). Madeiras Similares Ao Mogno (Swietenia Macrophylla King): Chave Ilustrada Para Identificação Anatõmica em Campo.

[9] Wiedenhoeft A (2011). Identification of Central American woods.

[10] Yin Y, Jiang X, Yuan L (2016). Identification manual of endangered and precious timber species common in trades. Biological division.

[11] Hermanson JC, Wiedenhoeft AC (2011). A brief review of machine vision in the context of automated wood identification systems. IAWA J.

[12] Cimpoi M, Maji S, Vedaldi A. Deep filter banks for texture recognition and segmentation. In: Proceedings of the IEEE conference on computer vision and pattern recognition; 2015. p. 3828–3836.

[13] Filho PLP, Oliveira LS, Nisgoski S, Britto AS (2014). Forest species recognition using macroscopic images. Mach Vis Appl.

[14] Krizhevsky A, Sutskever I, Hinton GE. Imagenet classification with deep convolutional neural networks. In: Pereira F, Burges CJC, Bottou L, Weinberger KQ, editors. Advances in neural information processing systems; 2012. p. 1097–105.

[15] Simonyan K, Zisserman A. Very deep convolutional networks for large-scale image recognition. 2014. CoRR arXiv:1409.1556.

[16] Szegedy C, Vanhoucke V, Ioffe S, Shlens J, Wojna Z. Rethinking the inception architecture for computer vision. 2015. CoRR arXiv:1512.00567.

[17] He K, Zhang X, Ren S, Sun J. Deep residual learning for image recognition. 2015. CoRR arXiv:1512.03385.

[18] Pan SJ, Yang Q (2010). A survey on transfer learning. IEEE Trans Knowl Data Eng.

[19] Russakovsky O, Deng J, Su H, Krause J, Satheesh S, Ma S, Huang Z, Karpathy A, Khosla A, Bernstein M, Berg AC, Fei-Fei L (2015). Imagenet large scale visual recognition challenge. Int J Comput Vis.

[20] Razavian AS, Azizpour H, Sullivan J, Carlsson S. CNN features off-the-shelf: an astounding baseline for recognition. 2014. CoRR arXiv:1403.6382.

[21] Helgason T, Russell SJ, Monro AK, Vogel JC (1996). What is mahogany? The importance of a taxonomic framework for conservation. Botan J Linnaeus Soc.

[22] Pennington TD, Styles BT, Taylor DAH (1981). Meliaceae. Flora Neotropica.

[23] LeCun Y, Boser B, Denker JS, Henderson D, Howard RE, Hubbard W, Jackel LD (1989). Backpropagation applied to handwritten zip code recognition. Neural Comput.

[24] Nair V, Hinton GE. Rectified linear units improve restricted boltzmann machines. In: Proceedings of the 27th international conference on machine learning. ICML’10; 2010. p. 807–14.

[25] Ioffe S. Szegedy C. Batch normalization: accelerating deep network training by reducing internal covariate shift. 2015. CoRR arXiv:1502.03167.

[26] Hinton GE, Srivastava N, Krizhevsky A, Sutskever I, Salakhutdinov R. Improving neural networks by preventing co-adaptation of feature detectors. 2012. CoRR arXiv:1207.0580.

[27] Buda M, Maki A, Mazurowski MA. A systematic study of the class imbalance problem in convolutional neural networks. 2017. CoRR arXiv:1710.05381. 10.1016/j.neunet.2018.07.011 30092410

[28] Kingma DP, Ba J. Adam: a method for stochastic optimization. 2014. CoRR arXiv:1412.6980.

[29] Chollet F, et al. Keras. GitHub. 2015. https://github.com/fchollet/keras

[30] Abadi M, Agarwal A, Barham P, Brevdo E, Chen Z, Citro C, Corrado GS, Davis A, Dean J, Devin M, Ghemawat S, Goodfellow IJ, Harp A, Irving G, Isard M, Jia Y, Józefowicz R, Kaiser L, Kudlur M, Levenberg J, Mané D, Monga R, Moore S, Murray DG, Olah C, Schuster M, Shlens J, Steiner B, Sutskever I, Talwar K, Tucker PA, Vanhoucke V, Vasudevan V, Viégas FB, Vinyals O, Warden P, Wattenberg M, Wicke M, Yu Y, Zheng X. Tensorflow: large-scale machine learning on heterogeneous distributed systems. 2016. CoRR arXiv:1603.04467.

[31] Gasson P (2011). How precise can wood identification be? Wood anatomy’s role in support of the legal timber trade, especially CITES. IAWA J.

[32] Esteva A, Kuprel B, Novoa RA, Ko J, Swetter SM, Blau HM, Thrun S (2017). Dermatologist-level classification of skin cancer with deep neural networks. Nature.

[33] Tang XJ, Tay YH, Siam NA, Lim SC. Rapid and robust automated macroscopic wood identification system using smartphone with macro-lens. 2017. CoRR arXiv:1709.08154.

[34] Zhou B, Khosla A, Lapedriza A, Oliva A, Torralba A. Learning deep features for discriminative localization. CVPR. 2016.

[35] Selvaraju RR, Das A, Vedantam R, Cogswell M, Parikh D, Batra D. Grad-cam: why did you say that? Visual explanations from deep networks via gradient-based localization. 2016. CoRR arXiv:1610.02391.

